# Sensitivity for multimorbidity: The role of diagnostic uncertainty of physicians when evaluating multimorbid video case-based vignettes

**DOI:** 10.1371/journal.pone.0215049

**Published:** 2019-04-10

**Authors:** Daniel Hausmann, Vera Kiesel, Lukas Zimmerli, Narcisa Schlatter, Amandine von Gunten, Nadine Wattinger, Thomas Rosemann

**Affiliations:** 1 Department of Psychology, University of Zurich, Zurich, Switzerland; 2 Department of Internal Medicine, University Hospital of Zurich, Zurich, Switzerland; 3 Institute of Primary Care, University Hospital of Zurich, Zurich, Switzerland; University of Zaragoza, SPAIN

## Abstract

**Background:**

Multimorbidity can be defined as the co-occurrence of two or more chronic medical conditions in one person. Within the diagnostic process, accurately detecting a multimorbid disease pattern still poses a major challenge for most physicians, and is known as a source of diagnostic uncertainty.

**Objective:**

We investigated, how sensitive, confident, and accurate physicians are in diagnosing multimorbid versus monomorbid patients.

**Methods:**

We created eight video case-based vignettes, which differed in type of morbidity (multimorbid versus monomorbid), field of medical specialization (somatic versus mental), and relatedness of underlying diseases (causally related versus unrelated). In total, 74 physicians (GPs, residents in an emergency department and psychiatrists) watched three to five randomly allocated video cases and had to generate suspected diagnoses at the end of each of three video sequences. Additionally, participating physicians rated subjective confidence for all mentioned diagnoses and for three sequences per case with the help of confidence profiles.

**Results:**

Altogether, physicians made a large number of accurate diagnoses (69%). Nevertheless, the overall number of underdiagnosed multimorbid cases (misses) was significantly higher (71%) than over-diagnosed monomorbid cases (false alarms) (7%).

**Discussion:**

According to Signal Detection Theory, GPs and psychiatrists both showed lower detection performance for medical cases that lay beyond their own field of specialization. Remarkably, residents show the highest sensitivity for multimorbid cases with an approximately identically detection performance d' slightly over 1 for both field of medical specialization (somatic and mental). Furthermore, higher uncertainty in diagnosing multimorbid cases is related to lower confidence especially at the beginning of a diagnostic process, as well as to unrelated and therefore probably rare disease pattern. Several limitations of the study and the video case-based vignettes are described within the discussion section.

**Conclusions:**

Physicians have to be sensitized for multimorbidity even more, and have to be taught in the prevalence of existing disease combinations. Communicating uncertainty with other specialists could be helpful when faced with a sometimes “fuzzy” pattern of symptoms.

## Introduction

The encouraging fact that life expectancy of people increased over the last few decades is strongly associated with cumulative medical issues [1]. A high and increasing incidence of multimorbidity is closely linked to ageing and growing populations of elderly people in the context of demographic change [2–5]. Multimorbidity can be defined as the co-occurrence of two or more chronic medical conditions in one person [6]. Nevertheless definitions are characterized by a high heterogeneity [7], but meanwhile mostly distinguished from the concept of comorbidity [4,8]. For instance, impacts of multimorbidity are found in a reduced quality of life of affected persons, the need for enhanced interdisciplinary collaboration among physicians, and increased financial burden for health care systems [9–13]. Despite its high prevalence rates and substantial effects on patients, physicians and health care systems, medical research is still focused on diagnosis and treatment of single diseases [14]. Knowing that multimorbidity is highly relevant in our present and future society, we raised the research question of how well physicians can handle multimorbid medical cases. Consequently, this article focuses on how sensitive, confident, and accurate physicians are in diagnosing multimorbid patients.

### Multimorbidity and medical decision making: A challenge

Multimorbidity in the context of medical decision making has been found to be challenging for physicians in several ways. For instance, GPs reported a lack of confidence and clinical competence when confronted with multimorbid patients and expressed a need for enhanced training and support [15].

Whereas classical medical decision making is based on finding the accurate diagnosis among several possible diagnoses (e.g. a decision for diagnosis A, B *or* C), multimorbid medical cases require the detection of an accurate combination of suspected diagnoses (e.g. A *and* C). Therefore, physicians, since they are aware of a high prevalence of multimorbidity, should demonstrate a certain degree of *sensitivity* at the beginning or during the diagnostic process and should be able to provide more accurate combinations of multimorbid diagnoses in the end. Besides thinking in a multi-optional way, physicians increasingly have to reflect and practice interdisciplinary cooperation, as well as use a more patient-centered approach [15,16]. Although multiple diagnoses are common in medical contexts, accurate guidelines for multimorbid diagnoses and treatments are missing [17,18].

The lack of universal guidelines may be a reason why diagnosing multimorbid patients is often accompanied with increased uncertainty in physicians [16,19,20]. Diagnostic certainty and confidence play an important role within the medical decision making process [21–25]. The term *confidence* describes “the belief, based on experience or evidence, that certain future events will occur as expected” (p. 706) [26]. Studies showed that confidence levels are increasing for final diagnoses during the medical decision making process, and are decreasing for diagnoses which are excluded in the end [25].

Another possible reason for physicians’ struggles when facing multimorbidity could be that multimorbid disease combinations do not always fall in one single field of expertise. In fact, interdisciplinary disease combinations, or more specifically the co-occurrence of somatic and mental diseases (e.g. depression/anxiety and chronic pain) in one person, are quite common [27–29]. Further studies showed that somatic diseases in mentally ill patients are often under-diagnosed in psychiatric care, whereas in primary care, mental disorders appear to be frequently under- or over-diagnosed [30–32]. And last, the role of relatedness of diseases remains unclear with regard to the diagnostic process. In some studies, researchers tend to refer to multimorbidity when talking about unrelated diseases, whereas referring to comorbidity in the case of causally related diseases [8].

For research into multimorbidity, the editorial of Mercer et al. claim new *shifts in design* [33]. Therefore, the novelty of our study is characterized by the simultaneous examination of three important variables: a) *disease pattern* (mono- versus multimorbidity), b) *disease combination* (somatic-somatic; somatic-mental; mental-mental), and c) *relatedness between two diseases* (causally related versus unrelated). These three variables have been examined within three different subgroups, representing physicians with different fields of specialization and experience: GPs, residents in an emergency department and psychiatrists. Regarding diagnostic processes, differences between GPs and even more experienced residents are well documented, e.g. for residents putting more effort into making a diagnosis [34], or representing a different attitude related to ethical issues [35].

### Research question and hypotheses

Based on frequent interdisciplinary disease combinations as well as regarding the reported general difficulties of physicians to diagnose and treat multimorbid patients accurately, the question arises whether physicians in different fields of specialization differ in their sensitivity in detecting multimorbid conditions. In our view, the Signal Detection Theory (SDT) is a useful theoretical and analytical reference tool to describe physicians’ sensitivity in terms of detection performance when diagnosing monomorbid versus multimorbid medical cases, using sensitivity measure *d’* [36]. Our first assumption states that physicians show lower sensitivity (*d’*) regarding multimorbidity in interdisciplinary medical cases compared to cases which fall into their own field of expertise. Regarding the role of causal relatedness of diseases in multimorbid cases as well as the impact of multimorbidity on confidence ratings within the diagnostic process, we secondarily presume that physicians’ confidence ratings are lower when confronted with multimorbid versus monomorbid medical cases. And third, we assume that physicians’ confidence ratings are higher when confronted with causally related multimorbid disease combinations in comparison with an unrelated pattern.

## Methods

### Participants

A total of 74 physicians in Switzerland took part in the study. Three groups of physicians with different fields of specialization were examined: 28 general practitioners (GPs) and 21 psychiatrists, as well as 25 residents working in an emergency department at the University Hospital of Zurich in the Division of Internal Medicine. Participating physicians were recruited through the Institute of Primary Care in Zurich (GPs), a public list of the Swiss Society for Psychiatry and Psychotherapy (psychiatrists), and with the help of the deputy director of the Division of Internal Medicine at the University Hospital in Zurich (residents). Inclusion criteria for GPs and psychiatrists were a minimum of five years of clinical experience and/or a specialist physician qualification and at least two years of clinical working experience for residents. In total, 63.5% of all participating physicians were male. The mean age of all participating physicians was 47.6 years (*SD* = 13.3), ranging from 26 to 71 years of age. Years of clinical experience differed between 2 and 45 years with an average of 19.5 years (*SD* = 13.2). Data collection took place between April and August 2013 and between June and July 2014 and was conducted by three graduate students (NS, AG, and NW) working at the University of Zurich, Department of Psychology, at that time. The whole data collection was conducted as part of three master theses. NS and AG collected data in parallel during the first period indicated for GPs and psychiatrists, whereas NW wrote her master thesis one year later about the residents in the emergency department, using the same core material as NS and AG.

### Materials

Realistic video case-based vignettes have been developed according to five steps [37,38], and used to portray a typical initial clinical interview situation. Therefore, eight video vignettes have been constructed in total, displaying symptoms of two monomorbid cases (one somatic and one mental), and six different disease combinations representing multimorbid cases with two underlying diseases each. Those six multimorbid cases followed the experimental design 3 (field of medical specialization: somatic-somatic; somatic-mental; mental-mental) x 2 (relatedness: causally related versus unrelated). Table 1 shows the characteristics of the eight video case-based vignettes in detail. All chosen multimorbid cases have been validated by experts in the fields of primary care and psychiatry for correctness and relatedness.

**Table 1:** Characteristics of all eight used video case-based vignettes.

**Monomorbid Case No.**	**Type of disease**		**Single disease**
Case 1 (M^s^)	somatic		food allergy
Case 2 (M^m^)	mental		obsessive compulsive-disorder (OCD)
**Multimorbid Case No.**	**Type of disease**	**Causal relatedness**	**Disease combination**
Case 3 (MM^ss-r^)	somatic & somatic	causally related	arterial hypertension & cardiac insufficiency
Case 4 (MM^ss-u^)	somatic & somatic	unrelated	arthrosis & hypothyreosis
Case 5 (MM^sm-r^)	somatic & mental	causally related	multiple sclerosis & depression
Case 6 (MM^sm-u^)	somatic & mental	unrelated	diabetes mellitus type 1 & posttraumatic stress disorder (PTSD)
Case 7 (MM^mm-r^)	mental & mental	causally related	psychotropic substance disorder & panic disorder
Case 8 (MM^mm-u^)	mental & mental	unrelated	social phobia & hypochondriac disorder

Note: M = monomorbid case; MM = multimorbid case

^s^ = somatic

^m^ = mental

^r^ = causally related

^u^ = unrelated.

Presented symptoms of the different diseases have been derived from ICD-10, which is used for the classification of mental and physical diseases on an international basis. One GP and one psychiatrist, both of whom had not been part of the participating physicians, evaluated the importance of all mentioned symptoms in the ICD-10 and derived four to six key symptoms for each disease. All symptoms used in the eight video vignettes are described in detail in S1 File.

The patient in each medical case was performed by the same trained actor, who was male and 41 years old, and from the “Carpe Mimos” actor agency in Zurich. He was dressed differently for each medical case and sometimes also wore make-up that matched with the reported symptoms (e.g., looking pale). Each medical case has been videotaped in three sequences with increasing informative content; each video sequence lasted between 16 and 48 seconds (*M* = 30 s, *SD* = 9 s). In the first video sequence, the actor entered a consulting room and sat down at the table, showing first indications of his disease(s) nonverbally, e.g. by limping or breathing heavily. The second video sequence continued the depicted clinical interview situation revealing two more symptoms for each monomorbid case respectively and three more symptoms in total for multimorbid cases by telling the imaginative physician about his medical condition. In the third video sequence, three more symptoms were articulated by the actor for both, monomorbid and multimorbid cases. The actor’s scripts of all used video vignettes can be found in S2 File. As we used these eight video case-based vignettes for the very first time, descriptive parameters of each medical case like diagnostic accuracy or evaluated difficulty etc. are displayed in detail in S1 Table.

Confidence profiles were used after each video sequence to assess physicians’ subjective confidence ratings regarding all mentioned diagnoses in a process-oriented way [25]. A confidence profile consists of several columns, one for each mentioned diagnosis and a corresponding rating scale for the subjective confidence levels (in percent) for one specific diagnosis at that point in time. For each mentioned diagnosis, participating physicians were requested to check one of 21 boxes of the rating scale, ranging from .00 to 1.00 in 5% intervals. For each video sequence physicians received a new plain confidence profile. Participating physicians were requested to rate their previously mentioned diagnoses as well as additional ones they gave in the respective sequence.

Demographic, case-related and general questions were assessed with multiple questionnaires. For the assessment of perceived realism of cases, the following item was used: “How realistic was this video case in your opinion?”. Physicians could answer on a five-level scale ranging from “not at all realistic” to “absolutely realistic”. Perceived difficulty of each case was assessed with the item “How difficult was it for you to make a diagnosis?” and could be answered on a five-level scale ranging from “very easy” to “very hard”.

In total, every participating physician filled in three confidence profiles and one case-related questionnaire per case, plus one short general questionnaire after evaluating all presented cases.

### Procedure

Participating physicians were shown the videotaped vignettes at their workplace in a quiet, inference-free room. First, the procedure and confidence profiles were explained. Subsequently, participating physicians were shown the video vignettes on a laptop. After the first, second and third video sequence for each case, they had to note possible diagnoses on the confidence profile and indicate their subjective confidence level for each mentioned diagnosis. Already mentioned diagnoses were transferred by one of the students to the next profile(s) and were evaluated by participating physicians again for the subsequent sequence(s). After having watched all three video sequences, a short case-related questionnaire had to be completed. Conclusively, a short general questionnaire was filled in before the participating physicians were thanked for their participation. As a reward for their participation, residents received a small bar of chocolate; GPs and psychiatrists could take part in a lottery with the chance of winning an iPad mini.

### Sample differences in procedure

None of the physicians were aware that we were investigating multimorbid versus monomorbid medical cases. Every GP and psychiatrist was presented a total of three different video case-based vignettes, depending on their specialization (type of disease). One vignette always showed a monomorbid case being presented as the first or second case. GPs were either presented the somatic or the mental monomorbid case, whereas psychiatrists were always shown the mental monomorbid case. Regarding multimorbid cases, GPs were shown two randomly assigned cases (with at least one somatic disease) from cases 3, 4, 5 and 6 (for the numbering of cases, see Table 1). Psychiatrists were also presented two multimorbid cases (with at least one mental disease), randomly chosen from cases 5, 6, 7 and 8. Because of their broader practical experience, residents were shown a total of five different vignettes in a randomly assigned order, both monomorbid cases, as well as all three unrelated multimorbid cases 4, 6 and 8.

### Analysis

Case-related descriptive statistics such as sample sizes, median case experience, numbers and distribution of given diagnoses, rated realism and evaluated difficulty of each presented case were calculated and are displayed in S1 Table.

#### Accuracy

Mentioned diagnoses of all presented cases have been listed and re-diagnosed by two of the authors (VK and DH) according to ICD-10, and afterwards checked by several medical students. Each listed diagnosis was then compared with the underlying diseases in the videos and classified either as accurate or inaccurate. Mentioned diagnoses were interpreted as accurate when they were situated on the same group classification of ICD-10 as the actual presented case. For mental disorders or disease combinations with at least one mental disorder, an accurate diagnosis additionally had to match with the class, e.g. the F40 –F48.

#### Detection performance

Signal Detection Theory (SDT) by Green and Swets [36] was used as an analytical reference of physicians’ detection performance. The SDT originally derived from the field of perceptional psychology and measures decision making under uncertainty. Four different types of responses can be differentiated and are used for the calculation of the measure *d’* which represents an estimation of detection performance or sensitivity regarding a certain stimulus. In the present study, the detection of multimorbid cases and its distinction from monomorbid cases were seen as a parallel to the detection of a stimulus and its distinction to random information in classical SDT. Concretely, we measured detection performance on group levels comparing at least two different medical cases, whereby one case was always multimorbid, the other one monomorbid. A physician’s diagnostic pattern could either be multi-optional, when corresponding with the underlying multimorbid case of a vignette (classified as a “hit”), or exclude multimorbidity while diagnosing one single disease, when corresponding with one of the two monomorbid cases (classified as a “correct rejection”).

#### Using confidence thresholds

As already introduced, participating physicians estimated numerical confidences for all suspected diagnoses, they have mentioned during the three sequences of a video case-based vignette. As it was not intended or possible for them to further explore a case or ask any further questions, all mentioned diagnoses have to be seen as preliminary diagnoses, rather than final diagnoses. This was the reason, why we made use of confidence thresholds for the classification, whether a specific diagnosis has to be regarded as accurate or not [39]. Franziska Bocklisch had translated linguistic terms of verbal probability expressions into estimated numerical values, using an empirical study design with 121 participants and calculating parametric fuzzy potential membership functions [40]. Most typical equivalents had resulted in a mean confidence value of .96 for the verbal expression “certain”, .84 for “very probable”, .75 for “quite probable”, .68 for “probable”, .51 for “possible”, .49 for “thinkable”, or .12 for “improbable”, etc. [40]. According to these equivalents, we assumed that a confidence level of .95 or higher (“certain”) is enough to determine a final diagnosis, whereas a level of .50 (“thinkable”) or even lower is not worth for further exploration within the diagnostic process. Including these confidence thresholds, a hit could be composed of three subtypes:

First, multimorbid diagnoses were named “totally accurate” when the confidence of an underlying accurate diagnosis for each single disease reached a required threshold of a minimum of .55 (including the verbal labels “possible”) [40] after the third sequence. Therefore, we assumed, that physicians would have pursued and deepened their diagnostic process in real medical practice, while keeping with corresponding verbal labels like “certain”, “very probable”, “quite probable”, “probable”, or “possible”, especially when confidence is too low to include or are too high to exclude a suspected diagnosis [39].

Secondly, a multimorbid diagnosis was termed “partially accurate” when it was defined by a combination of an accurate diagnosis with a minimal confidence of .55 and an inaccurate diagnosis with a reported minimal confidence of .95 after sequence three.

Finally, also “totally inaccurate” diagnoses were included in the category “hit” that consisted of two inaccurate diagnoses with a minimal confidence level of .95 each after sequence three.

In addition, physicians could also make mistakes by diagnosing as multimorbid in an underlying monomorbid case vignette or assessing a multimorbid video vignette as a single disease. Over-diagnosing multimorbidity was classified as a “false alarm” whereas under-diagnosing was indicated as a “miss”. This classification of responses was only applied when a physicians’ confidence rating after the third video sequence was at least .55 for diagnoses previously classified as accurate. Diagnoses which have been classified as inaccurate had to be reported with a confidence of at least .95 after the third sequence. If a physician did not reach the required certainty thresholds mentioned above for any suspected diagnosis, the category “no diagnosis” was assigned. This category was not used for calculations and is therefore not presented in Table 2. Thus, physicians’ responses could be arranged in a fourfold table (see Table 2).

**Table 2:** The fourfold table of possible diagnostic responses.

	Underlying diagnosis (as per video case-based vignette)	
	Multimorbidity	Monomorbidity
**Type of diagnosis made by a physician**		
***Multimorbid***	**hit**	false alarm
***Monomorbid***	miss	**correct rejection**

Note: Terms “hit”, “false alarm”, “miss” and “correct rejection” are derived from Signal Detection Theory of Green & Swets (1966).

#### Sensitivity measure (d’)

For the estimation of physicians’ sensitivity, the relative amounts of “hits” and “false alarms” in the physicians’ diagnostic responses were used. The measure *d’* was calculated by subtracting the z-transformed false alarm rate from the z-transformed hit rate. The more sensitively the physicians performed in detecting multimorbidity in the different medical cases the greater the value of *d’*.

In order to examine our first hypothesis, group comparisons between all three groups of physicians were conducted, focusing on different types of multimorbidity (somatic and somatic, mental and mental, or mixed). For hypotheses two and three we included only confidence ratings of accurately mentioned diagnoses irrespective of thresholds or SDT. All analyses were performed using Microsoft Excel and IBM SPSS Statistics 23 for Windows, and all tests of significance employed [α] = .05. Furthermore, effect sizes (*d*) were calculated according to Cohen.

## Results

In summary, 74 physicians performed a total of 269 medical cases, from which 98 were monomorbid and 171 were multimorbid. Of 1027 reported diagnoses in total, physicians mentioned 1 to 10 suspected diagnoses per person and case over the three video sequences (*M* = 3.82; *SD* = 1.79) (see also S3 File).

Physicians showed good accuracy for both monomorbid cases. According to the chosen confidence thresholds (see analysis section), 75.0% resulted in an accurate diagnosis of obsessive-compulsive disorder (case 2), and only one physician (1.7%) in an inaccurate monomorbid diagnosis, four (6.7%) in an over-diagnosis (partly accurate multimorbid diagnosis), and 10 (16.7%) didn’t reach the threshold for any diagnosis (no diagnosis). For food allergy (case 1) only 31.6% resulted in an accurate diagnosis, three (7.9%) in an inaccurate monomorbid diagnosis, and none in an over-diagnosis. The majority of 60.6% didn’t reach the threshold for any diagnosis (no diagnosis) for case 1.

The percentage of no diagnosis for the multimorbid cases (3 to 8) was within the range of 10% to 28%, whereas on average 22.3% mentioned diagnostic pattern resulted in a hit (15.8% totally accurate; 5.3% partially accurate; and only 1.2% totally inaccurate). The majority resulted in an under-diagnosis, which means that on average 55.5% of the physicians missed one of the two diagnoses (for details see S1 Table).

**Fig 1:**
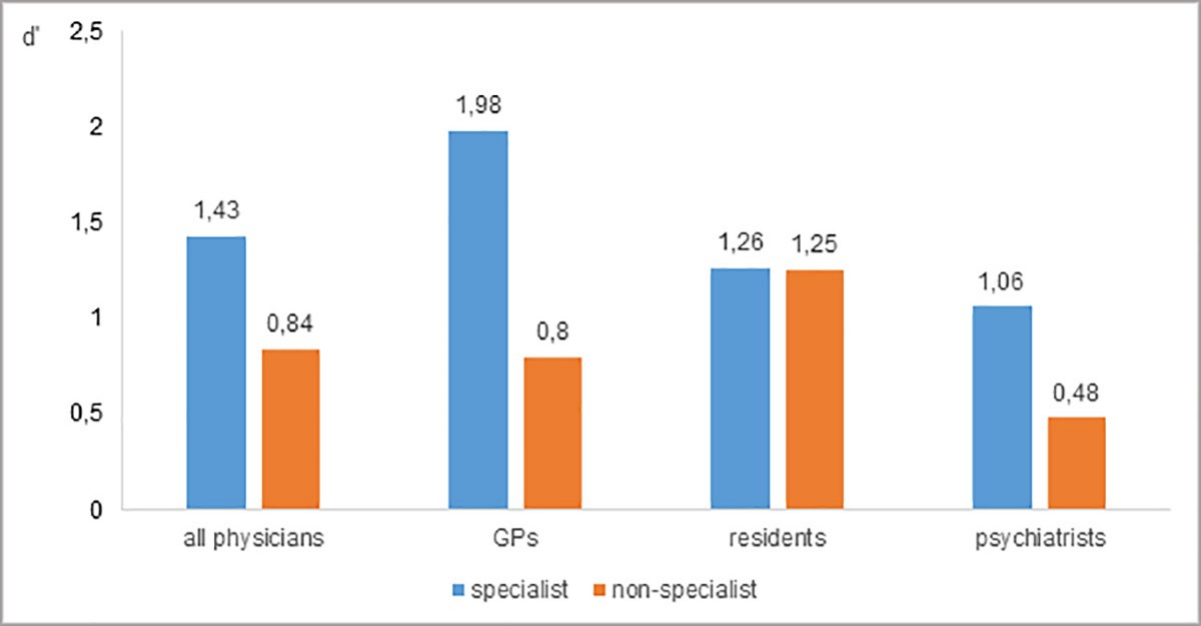
Detection performance (*d’*) of physicians for cases from different fields of specialization.

As illustrated in Fig 1, the overall mean *d’* reached a higher value for multimorbid medical cases that fall within physicians’ own field of expertise (specialist) with a mean detection performance (*d’*) of 1.43 for all physicians compared to interdisciplinary (non-specialist) medical cases with an average *d’* of 0.84. This pattern with a *d’* larger than 1 for disease combinations in the own medical field of specialization and a d’ lower than 1 for non-specialist cases can be found for GPs as well as for psychiatrists but not for residents. Residents show an approximately identically detection performance *d’* slightly over 1 (see Fig 1). Therefore our first assumption of lower sensitivity for interdisciplinary multimorbid medical cases could be confirmed for GPs and psychiatrists, but not for residents.

**Fig 2:**
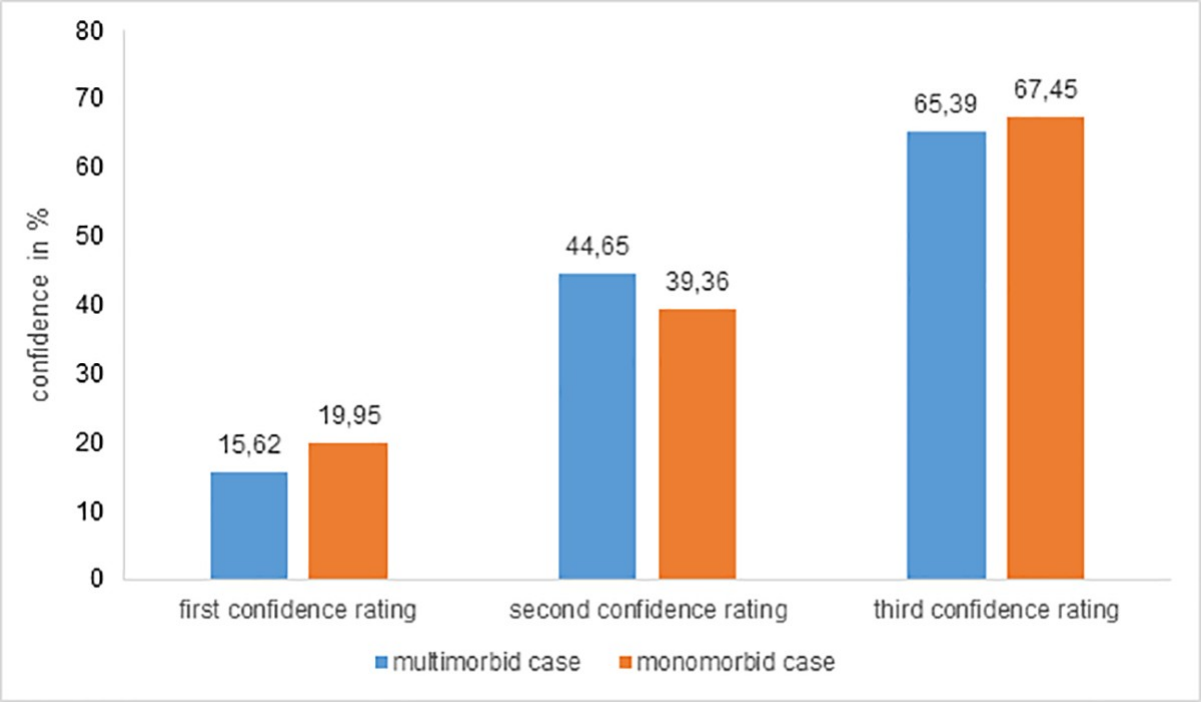
Mean confidence ratings of all physicians for multimorbid and monomorbid cases for all three successive video sequences.

As expected, confidence ratings for accurately suspected diagnoses are rising from the first to the third video sequence, for monomorbid as well as for multimorbid cases (see Fig 2). Nevertheless, rating differences were not significantly higher for multimorbid cases as assumed in our second hypothesis. Whereas on average physicians’ confidence ratings were by trend higher for monomorbid cases, ratings were even lower after the second sequence. However, a closer look at group differences after the first video sequence revealed statistically significant lower confidence ratings with small effect sizes at least for GPs (*M*_*MM*_ = .10; *SD*_*MM*_ = .22, versus *M*_*M*_ = .21; *SD*_*M*_ = .26; *t* = 1.95, *df* = 38, *p* = .030, one-tailed; *Cohen’s d* = 0.48; *CI* = [0.037–0.917]) and psychiatrists (*M*_*MM*_ = .21; *SD*_*MM*_ = .31, versus *M*_*M*_ = .33; *SD*_*M*_ = .34; *t* = 1.72, *df* = 88, *p* = .044, one-tailed; *Cohen’s d* = 0.37; *CI* = [0.055–0.800]) to the disadvantage of multimorbid cases. This means, that even without having spoken to the patient–but having seen the patient entering the room–resulted in higher confidence ratings for monomorbid than for multimorbid issues of accurately mentioned diagnoses.

Regarding confidence ratings for accurately mentioned diagnoses for each multimorbid video case, ratings were in principal higher when confronted with causally related disease combinations in comparison to unrelated combinations. For all three causally related video case-based vignettes, confidence ratings at the end of sequence three were comparable high with .79 for cases 5 and 7, and .76 for case 3, which all corresponds to a verbal label of (“quite probable”), compared to the numerical translation of linguistic terms [40]. For unrelated cases 4 (.57) and 6 (.56), confidence ratings were on average considerably lower, which corresponds both to a verbal label of (“possible”) [40]. Only the last unrelated case 8 resulted in a higher confidence rating of .76 (“quite probable”). Overall we found a statistically significant difference between confidence ratings of all three causally related cases (*M* = .78; *SD* = .26) and all those for unrelated multimorbid cases (*M* = .61; *SD* = .31) as assumed (*t* = 4.25, *df* = 125, *p* = .000, one-tailed; *Cohen’s d* = 0.56; *CI* = [0.272–0.849]). Physicians showed more uncertainty for unrelated than for causally related multimorbid cases. No significant group differences between participating physicians have been found.

## Discussion

Even though the prevalence of multimorbid medical cases is increasing steadily, less is known about how accurate, confident and sensitive physicians are when diagnosing multimorbid patients. Our experimental study design with eight video case-based vignettes revealed some specific factors that make diagnosing multimorbid medical cases challenging for physicians. First of all, GPs and psychiatrists showed worse detection performance for cases that did not fully fall into their own medical field of specialization, as for example for mixed multimorbid cases (somatic and mental) [30–32]. Second, especially at the beginning of the diagnostic process, GPs and psychiatrists are significantly less certain about accurate suspected diagnoses, when confronted with a multimorbid patient. And third, physicians express significantly less certainty, if two underlying diseases were unrelated [33].

### Accuracy

Altogether, physicians made a large number of accurate diagnoses (69%). Nevertheless, the overall number of underdiagnosed multimorbid cases (misses) was significantly higher (71%) than over-diagnosed monomorbid cases (false alarms) (7%) [30–32]. Furthermore, we observed a substantial number of no diagnoses made for multimorbid cases, because of the fact that a lot of accurate diagnoses have been mentioned with very low confidence ratings at the end of the video sequences and therefore have fallen below the threshold.

### Confidence

Significant differences in confidence ratings between accurately mentioned multimorbid und monomorbid diagnoses have been found for the visual diagnosis at the beginning of the diagnostic process. After the last video sequences, physicians ended up with a comparable rating (“probable”) [40] for monomorbid and multimorbid cases after having seen all symptoms. While confidence ratings for accurate diagnoses are generally increasing over the three sequences, ratings are decreasing for inaccurately suspected diagnoses. Increasing confidence levels for final diagnoses and decreasing levels for finally excluded suspected diagnoses correspond to an evidence accumulation process described in literature [41] as well as have been observed in real diagnostic processes [25].

Within multimorbid cases, the relatedness of the underlying diseases seems to be very important for the subjective certainty of physicians. When confronted with two unrelatedly existing diseases in our study, significantly less confidence is given to accurate diagnoses (“possible”) [40], than for those of related ones (“quit probable”) [40]. One reason for reduced confidence ratings could be, that unrelated multimorbid cases are rarer than related ones [42,43].

### Sensitivity

For expressing detection performance of physicians, we calculated the sensitivity measure *d’* according to Signal Detection Theory (SDT) [36] as the relation of the relative amount of hits and false alarms on a group level. As a result, all three groups of physicians showed satisfactory detection performance for medical cases that lay within their own field of specialization (*d*’ > 1). GPs showed the highest detection performance (*d*’ = 2). On the contrary, GPs and psychiatrists both showed lower detection performance (*d*’ < 1) for medical cases that lay beyond their own field of specialization. Somehow atypical, residents in our study showed higher detection performance for non-specialist cases (*d*’ = 1.25) and higher confidence ratings for multimorbidly suspected diagnoses at the beginning of the diagnostic process (visual diagnosis), compared to GPs or psychiatrists. One possible explanation for these results could be that residents in an emergency department are accustomed to seeing heterogeneous disease pattern more often, which do not only fall into their own field of specialization. Furthermore, most of our residents could have been trained in diagnosing multimorbid patients or having anticipated multimorbid cases in our study, because multimorbidity is one of the research foci of the department they are working for.

The high percentage of “no diagnoses” made within some cases (e.g. case 4 and 6), seems to be a relevant category for detection performance too, because it may well express physicians’ diagnostic uncertainty. In real clinical practice, rating low confidences for several suspected diagnoses could also end up in a wait-and-see strategy, especially as known for GPs [44,45].

Overall, sensitivity has to be seen as a prerequisite [46] for detecting, diagnosing, and handling multimorbidity; particularly for looking at presented pattern of symptoms more carefully, for clarifying additive symptoms in more detail, as well as for thinking about and opting for appropriate medical treatment strategies.

### Limitations

According to the explicit ratings of physicians, all eight video case-based vignettes have been *perceived as realistic* in principle, ranging from 3.2 to 3.8 on a scale from 1 (“not at all realistic”) to 5 (“absolutely realistic”). Also *perceived difficulty* was estimated as moderate (2.2 to 3.7), while the scale ranges from 1 (“very easy”) to 5 (“very hard”) (for details see S1 Table). According to *accuracy*, the discrepancy between correctly diagnosing both monomorbid cases was high. Detecting obsessive-compulsive disorder (OCD, case 2) accurately was more frequent (75%) than food allergy (case 1) (32%). Furthermore there is a high rate of no diagnosis made for food allergy (61%), compared to OCD (17%).

As a fact, the selection of multimorbid cases had to be limited in general scope and kind of combinations within our experimental design. Therefore, several potential effects of single diseases or particular combinations thereof cannot be estimated appropriately, depending on the different case experiences of the participating physicians. Furthermore, the patient in each video case-based vignette was performed by the same trained actor (only varying clothing and make-up), which, in a positive sense, generated no experimental effects (of potentially different actors). Nevertheless, it can be questioned if gender and age of the patient was ideally fitting for all multimorbid cases (e.g. diabetes mellitus type 1). In question of representativeness, one has to consider that our three samples of GPs, psychiatrists in private practice, and residents working in an emergency unit are characteristic for their medical working fields in the German speaking part of Switzerland, and no particular selection biases have been observed (apart from the residents). Nevertheless, as an outlook, our methodological approach should be expanded to other countries and should include larger samples in future studies.

None of the physicians were aware that we aimed to investigate multimorbid versus monomorbid medical cases, which has to be seen as a benefit of our experimental design too. And this is the reason, why we did not explicitly ask them for a final monomorbid or multimorbid diagnosis after each video. Moreover, most physicians would not have been able to determine a final diagnosis, because they had no possibility to interact with the patient or actively ascertain further information about him, as opposed to real clinical practice. Therefore, we had to estimate final diagnoses post-hoc according to the physicians’ confidence ratings at the third and last video sequence according to a confidence threshold. Those chosen confidence thresholds were plausible and well defined according to the equivalent of verbal probability expressions in literature [40] indeed, but criticizable in principle. Furthermore and in general, subjective confidence ratings of participating physicians have to be interpreted carefully, because there is no validated measuring scale of probabilities [25].

In our view, SDT is a useful theoretical and analytical reference tool to describe physicians’ detection performance when diagnosing multimorbid versus monomorbid medical cases, using the sensitivity measure *d’* [36]. But within our study, calculating detection performance was only possible on a group level, and clear guidelines and recommendations are missing for the interpretation of the degree of *d’*, its ranges, or even extents of group differences. As an error-free behavior is not provided within SDT, sometimes *d’* could only be calculated by approximation–as in some cases physicians showed no false alarms. Furthermore, all “no diagnosis” had to be excluded from any calculations according to SDT.

### Future research directions and clinical implications

Video case-based vignettes provide a good opportunity to investigate physicians’ diagnostic processing and handling of multimorbidity in the areas of research, education and clinical training. Including confidence profiles [25] allows assessing courses of confidence ratings and therefore the visualization of physicians’ subjective certainty about suspected diagnoses at each time point during the diagnostic process. With the help of these methodological tools, investigating factors that enhance or reduce diagnostic uncertainty would be a subsequent research issue to deepen, as for example the moderating effect of the relatedness of multimorbid diseases. Furthermore, the mechanisms (stopping rules) of determining a final diagnosis should be scientifically explored in more detail, as making a final diagnosis can be seen as transition from diagnosis to treatment [47].

Multimorbid disease pattern do not follow the border of physicians’ fields of medical specialization. Physicians have to be sensitized for multimorbidity even more, and have to be taught in the prevalence of existing disease combinations within and as well as outside their medical field of specialization [15]. What makes it difficult to diagnose a multimorbid medical case? Typically, patients are not aware of an underlying combination of distinct diseases [48]. Therefore, during the anamnesis patients may report symptoms in an incoherent order (see script of our eight video case-based vignettes in S2 File). Furthermore, some of the symptoms might stand in an interaction to other symptoms and therefore are less or differently present by contrast of an apparent single disease. Based on this atypical and sometimes “fuzzy pattern” of symptoms, it seems not easy for physicians to filter out two or more distinct medical diseases. Furthermore, multimorbidity often needs an enhanced interdisciplinary collaboration among physicians, because a lot of disease combinations go beyond the specialization of a single physician [15]. Suspected disease combinations have to be detected first by a GP, resident, or psychiatrist, than further elaborated, and finally often discussed and ensured by another specialist. “Communicating about the uncertainty” [49] (e.g. with the estimated subjective probability for a suspected diagnosis at a specific point in time) can help to unfold and express that there could be more than a single disease, to try to achieve more evidence with further diagnostic activity, and finally reach a sufficient level and clarity and certainty to diagnose it as a separate disease. Altogether, research for multi-optional decision making, especially in diagnosing multimorbidity, is only just beginning. And apart from diagnostics, the treatment of multimorbidity remains the second unresolved challenge of a physician [50].

## Conclusions

Multimorbidity continues to represent a major challenge within the diagnostic process. Our study revealed that detecting and diagnosing multimorbid medical cases seems to be that the less related the underlying diseases are, the more difficult detection and diagnosis are for physicians. Therefore, it is beneficial if future investigations explore and describe the incidence and relatedness of disease combinations and teach this knowledge to physicians. Finally, communicating about the uncertainty of suspected diagnoses with other specialists could help in further exploring and not missing a multimorbid disease pattern within patients.

## Supporting information

S1 File Overview over symptoms. Overview over presented symptoms within video case-based vignettes, broken down by individual diseases or disorders.
(DOCX)

S2 File Actor’s scripts. Actor’s scripts for the eight video case-based vignettes and all three sequences (S1 to S3).
(DOCX)

S3 File Raw data file of a total of 1027 mentioned diagnoses. Confidence ratings and kind of diagnoses for all participating physicians and cases (see also legend in separate worksheet).
(XLSX)

S4 File Consent form. Consent form for all participating physicians to read and to sign.
(PDF)

S5 File Instruction. Written instructions for watching the video case-based vignettes, filling in suspected diagnoses and confidence ratings in a confidence profile after each sequence, as well as filling in short case-related questionnaires and finally a short general questionnaire.
(PDF)

S6 File Confidence profile. Empty template for filling in suspected diagnoses and confidence ratings after each sequence of a video.
(PDF)

S7 File Case-related questionnaire. Empty sheet for filling in additional information about suspected diagnoses, reference of patient, difficulty of diagnosis, and missing additional information after each video.
(PDF)

S8 File General questionnaire. Empty sheet for filling in additional information about the difficulty of making a diagnosis, case experience and difficulty, and missing additional information at the end of participation.
(PDF)

S1 Table Overview over cases. Descriptive statistics for all eight video case-based vignettes (for abbreviations see Table 1).
(DOCX)

## References

[1] Divo MJ, Martinez CH, Mannino DM. Ageing and the epidemiology of multimorbidity. Eur Respir J. 2014;44: 1055–1068. 10.1183/09031936.00059814 25142482 PMC4918092

[2] Melis R, Marengoni A, Angleman S, Fratiglioni L. Incidence and predictors of mutlimorbidity in the elderly: A population-based longitudinal study. PLOS ONE. 2014;9(7): e103120 10.1371/journal.pone.0103120 25058497 PMC4109993

[3] Murphy E. Case management and community matrons for long term conditions. BMJ. 2004;329(7477): 1251–1252. 10.1136/bmj.329.7477.1251 15564235 PMC534431

[4] Van den Akker M, Buntinx F, Metsemakers JFM, Roos S, Knottnerus JA. Multimorbidity in general practice: Prevalence, incidence, and determinants of co-occurring chronic and recurrent diseases. J Clin Epidemiol. 1998;51(5): 367–375. 9619963 10.1016/s0895-4356(97)00306-5

[5] Scheidt-Nave C, Richter S, Fuchs J, Kuhlmey A. Challenges to health research for aging populations using the example of „multimorbidity“. Bundesgesundheitsbl. 2010;53(5): 441–450. 10.1007/s00103-010-1052-9 20376419

[6] Bopp M, Holzer BM. Prevalence of multimorbidity in Switzerland: Definition and data sources. Praxis. 2012;101(25): 1609–1613. 10.1024/1661-8157/a001143 23233098

[7] Marengoni A, Angleman S, Melis R, Mangialasche F, Karp A, Garmen A, et al Aging with multimorbidity: A systematic review of the literature. Ageing Res Rev. 2011;10(4): 430–439. 10.1016/j.arr.2011.03.003 21402176

[8] Beyer MM, Otterbach IM, Erler AM, Muth CM, Gensichen JM, Gerlach FM. Multimorbidity in General Practice Part I: A pragmatic definition, epidemiology, prerequisites of care. Z Allg Med. 2007;83(8): 310–315.

[9] Anderson G, Horvath J. The growing burden of chronic disease in America. Public Health Rep. 2004;119(3): 263–270. 10.1016/j.phr.2004.04.005 15158105 PMC1497638

[10] Roy-Byrne PP, Davidson KW, Kessler RC, Asmundson GJG, Goodwin RD, Kubzansky L, et al Anxiety disorders and comorbid medical illness. Gen Hosp Psychiatry. 2008;30(3): 208–225. 10.1016/j.genhosppsych.2007.12.006 18433653

[11] Hodek JM, Ruhe A, Greiner W. Multimorbidity and health-related quality of life among elderly persons. Bundesgesundheitsbl. 2009;52(12): 1188–1201. 10.1007/s00103-009-0974-6 20012567

[12] Agborsangaya CB, Lau D, Lathinen M, Cooke T, Johnson JA. Health-related quality of life and healthcare utilization in multimorbidity: Results of a crosssectional survey. Qual Life Res. 2013;22(4): 791–799. 10.1007/s11136-012-0214-7 22684529

[13] Felker B, Yazel JJ, Short D. Mortality and medical comorbidity among psychiatric patients: A review. Psychiatr Serv. 1996;47(12): 1356–1363. 10.1176/ps.47.12.1356 9117475

[14] Boyd CM, Darer J, Boult C, Fried LP, Boult L, Wu AW. Clinical practice guidelines and quality of care for older patients with multiple comorbid diseases: Implications for pay for performance. JAMA. 2005;294(6): 716–724. 10.1001/jama.294.6.716 16091574

[15] Smith SM, O’Kelly S, O’Dowd T. GPs’ and pharmacists’ experiences of managing multimorbidity: A “Pandora’s box’. Br J Gen Pract. 2010;60(576): e285–294. 10.3399/bjgp10X514756 PMC2894403 20594430

[16] Ickowicz E. Guiding principles for the care of older adults with multimorbidity: An approach for clinicians. J Am Geriatr Soc. 2012;60(10): E1–25. 10.1111/j.1532-5415.2012.04188.x 22994865 PMC4450364

[17] Blozik E, Dubben HH, Wagner HO, Scherer M. Comorbidity in medical guidelines: Comparison of the current state, epidemiologic models and expert opinion. ZEFQ. 2014;108(4): 219–228. 10.1016/j.zefq.2014.02.001 24889711

[18] Muth C, Kirchner H, van den Akker M, Scherer M, Glasziou PP. Current guidelines poorly address multimorbidity: Pilot of the interaction matrix method. J Clin Epidemiol. 2014;67(11): 1242–1250. 10.1016/j.jclinepi.2014.07.004 25216898

[19] Roland M, Paddison C. Better management of patients with multimorbidity. BMJ. 2013;346: f2510 10.1136/bmj.f2510 23641032

[20] Sinnott C, Mc Hugh S, Browne J, Bradley C. GPs’ perspectives on the management of patients with multimorbidity: Systematic review and synthesis of qualitative research. BMJ Open. 2013;3(9): e003610 10.1136/bmjopen-2013-003610 24038011 PMC3773648

[21] Kostopoulou O. Uncertainty in medical decisions In: Kattan MW, editor. Encyclopedia of Medical Decision Making. Thousand Oaks (CA): Sage Publications; 2009 pp. 1157–1160.

[22] Lutfey KE, Link CL, Marceaau LD, Grant RW, Adams A, Arber S, et al Diagnostic certainty as a source of medical practice variation in coronary heart disease: Results from a cross-national experiment of clinical decision making. Med Decis Making. 2009;29(5): 606–618. 10.1177/0272989X09331811 19470719 PMC3755454

[23] Cosby K. The role of certainty, confidence, and critical thinking in the diagnostic process: Good luck or good thinking? Acad Emerg Med. 2011;18(2): 212–214. 10.1111/j.1553-2712.2010.00979.x 21314782

[24] Jackson SA, Kleitman S. Individual differences in decision-making and confidence: Capturing decision tendencies in a fictitious medical test. Metacogn Learn. 2014;9(1): 25–49.

[25] Hausmann D, Zulian C, Battegay E, Zimmerli L. Tracing the decision-making process of physicians with a Decision Process Matrix. BMC Med Inform Decis Mak. 2016;16: 133 10.1186/s12911-016-0369-1 27756369 PMC5070075

[26] Siegrist M, Earle TC, Gutscher H. Test of a trust and confidence model in the applied context of Electromagnetic Field (EMF) risks. Risk Analysis. 2003;23(4): 705–716. 12926564 10.1111/1539-6924.00349

[27] Agborsangaya CB, Lau D, Lahtinen M, Cooke T, Johnson JA. Multimorbidity prevalence and patterns across socioeconomic determinants: A cross-sectional survey. BMC Public Health. 2012;12(1): 201–208. 22429338 10.1186/1471-2458-12-201 PMC3353224

[28] Andrade LH, Benseñor IM, Viana MC, Andreoni S, Wang YP. Clustering of psychiatric and somatic illnesses in the general population: Multimorbidity and socioeconomic correlates. Braz J Med Biol Res. 2010;43(5): 483–491. 20379689 10.1590/s0100-879x2010007500024

[29] Jakovljević M, Ostojić L. Comorbidity and multimorbidity in medicine today: Challenges and opportunities for bringing separated branches of medicine closer to each other. Psychiatr Danub. 2013;25(1): 18–28. 23806971

[30] Röhr F, Schürmann J, Tölle R. Körperliche Untersuchungen bei psychisch Kranken. Dtsch Ärztebl. 1996;93: A1899–1903.

[31] Hensler S, Wiesemann A. Discrediting studies in German general practices–Or: How to increase prevalence of diseases and the need for drug treatment? Z Allg Med. 2003;79(12): 579–585.

[32] Sielk M, Abholz HH. Why do General Practitioners characterize patients as more depressive than psychiatrists do? Z Allg Med. 2005;81(11): 486–490.

[33] Mercer SW, Gunn J, Bower P, Wyke S, Guthrie B. Managing patients with mental and physical multimorbidity: Changes are needed in policy, research, and practice. BMJ. 2012;345: e5559 10.1136/bmj.e5559 22945951

[34] Wang M, Wild S, Hilfiker G, Chmiel C, Sidler P, Eichler K, et al Hospital-integrated general practice: A promising way to manage walk-in patients in emergency departments. J Eval Clin Pract. 2014;20(1): 20–6. 10.1111/jep.12074 24033413

[35] Krütli P, Rosemann T, Törnblom KY, Smieszek T. How to fairly allocate scarce medical resources: Ethical argumentation under scrutiny by health professionals and lay people. PLOS ONE. 2016;11(7): e0159086 10.1371/journal.pone.0159086 27462880 PMC4963105

[36] Green DM, Swets JA. Signal detection theory and psychophysics New York (NY): Wiley; 1966.

[37] van Vliet LM, Hillen MA, van der Wall E, Plum N, Bensing JM. How to create and administer scripted video-vignettes in an experimental study on disclosure of a palliative breast cancer diagnosis. Patient Educ Couns. 2013;91: 56–64. 10.1016/j.pec.2012.10.017 23219482

[38] Hillen MA, van Vliet LM, de Haes HCJM, Smets EMA. Developing and administering scripted video vignettes for experimental research of patient-provider communication. Patient Educ Couns. 2013;91: 295–309. 10.1016/j.pec.2013.01.020 23433778

[39] Hausmann D, Läge D. Sequential evidence accumulation in decision making: The individual desired level of confidence can explain the extent of information acquisition. Judgm Decis Mak. 2008;3(3): 229–243.

[40] Bocklisch F. The vagueness of verbal probability and frequency expressions. Int J Adv Comput Sci. 2011;1: 52–57.

[41] Busemeyer JR, Townsend JT. Decision Field Theory: A dynamic-cognitive approach to decision making in an uncertain environment. Psychol Rev. 1993;100(3): 432–459. 8356185 10.1037/0033-295x.100.3.432

[42] Schäfer I, Kaduszkiewicz H, Wagner HO, Schön G, Scherer M, van den Busche H. Reducing complexity: A visualisation of multimorbidity by combining disease clusters and triads. BMC Public Health. 2014;14: 1285 10.1186/1471-2458-14-1285 25516155 PMC4301832

[43] Violan C, Foguet-Boreu Q, Flores-Mateo G, Salisbury C, Blom J, Freitag M, et al Prevalence, determinants and patterns of multimorbidity in primary care: A systematic review of observational studies. PLOS ONE. 2014;9(7): e102149 10.1371/journal.pone.0102149 25048354 PMC4105594

[44] Waldmann UM, Gulich M, Stabenow U, Zeitler HP. A complex process: Decision-making in general practice: 117 structured case analyses. Wien Med Wochenschr. 2006;156(23): 633–643. 17211769 10.1007/s10354-006-0352-z

[45] Wübken M, Oswald J, Schneider A. Dealing with diagnostic uncertainty in general practice. ZEFQ. 2013;107(10), 632–637. 10.1016/j.zefq.2013.10.017 24315334

[46] Rest JR. Background: Theory and research In: Rest JR, Narvaez D, editors. Moral development in the professions: Psychology and applied ethics. Hillsdale (NJ): Taylor & Francis; 1994 pp. 1–26.

[47] Djulbegovic B, Elqayam S, Reljic T, Hozo I, Miladinovic B, Tsalatsanis A, et al How do physicians decide to treat: An empirical evaluation of the threshold model. BMC Med Inform Decis Mak. 2014;14(1): 47–56. 24903517 10.1186/1472-6947-14-47 PMC4055375

[48] Gibbons CJ, Kenning C, Coventry PA, Bee P, Bundy C, Fisher L, et al Development of a multimorbidity illness perceptions scale (MULTIPLEeS). PLOS ONE. 2013;8(12): e81852 10.1371/journal.pone.0081852 24376504 PMC3869652

[49] Fischhoff B, Davis AL. Communicating scientific uncertainty. PNAS. 2014;111(4): 13664–13671. 25225390 10.1073/pnas.1317504111 PMC4183175

[50] Markun S, Holzer BM, Rodak R, Kaplan V, Wagner C, Battegay E, et al Therapeutic conflicts in emergency department patients with multimorbidity: A cross-sectional study. PLOS ONE. 2014;9(10): e110309 10.1371/journal.pone.0110309 25310005 PMC4195608

