# Supplementary material for: Sensitivity for multimorbidity: The role of diagnostic uncertainty of physicians when evaluating multimorbid video case-based vignettes
Source: PLoS One. 2019 Apr 10;14(4):e0215049. doi: 10.1371/journal.pone.0215049 (PMC6457556; doi:10.1371/journal.pone.0215049)
Supplement: S1 File — Overview over presented symptoms within video case-based vignettes, broken down by individual diseases or disorders. (DOCX) [file pone.0215049.s001.docx]

**S1 File**

Overview over presented symptoms within video case-based vignettes, broken down by individual diseases or disorders.

| **Disease** (part of case x) | **Incorporated symptoms** |
| --- | --- |
| **food allergy** (case 1) | - skin rash / redness - itchy skin - gastrointestinal problems - symptoms 1 to 2 times a week - swollen tongue / lips - difficulty in breathing |
| **obsessive-compulsive disorder (OCD)** (case 2) | - obsessive thoughts / compulsive acts are disturbing - > 6 months - disturbance of occupational activity - patient realizes his impulses and thoughts - attempted resistance is failing - feelings of shame |
| **arterial hypertension** (case 3) | - pulsation in the head under strain - headaches - red head - dizziness |
| **cardiac insufficiency** (case 3) | - shortness of breath - swollen legs - has to remain upright once asleep to breathe - retention of fluid |
| **arthrosis** (case 4) | - swelling - pain after sport activity - “start-up” pain (when getting up in the morning) - limited mobility / loss of function |
| **hypothyreosis** (case 4) | - tired, listless, slowed down - pale skin - weight gain - increased sensitivity to cold |
| **multiple sclerosis** (case 5) | - degradation in stressful situations - visual disturbances / eye complaints - muscular paralysis or weakness / stiffness in one leg - paresthesia |
| **depression** (case 5) | - mood fluctuation - weight loss - sleep disturbance - listlessness |
| **diabetes mellitus type 1** (case 6) | - strong thirst - tiredness - weight loss - constant desire to void |
| **posttraumatic stress disorder (PTSD)** (case 6) | - traumatic experience - flashbacks of trauma - increased vigilance - > 1 month |
| **psychotropic substance disorder**  (case 7) | - depersonalization - increased consumption - potential hyperactivity - damage to health (damage to nasal septum) |
| **panic disorder** (case 7) | - tachycardia - feelings of suffocation - fear of another panic attack - fear of going mad |
| **social phobia** (case 8) | - fear of giving a talk - hand trembling - fear of vomiting in public - avoidance of phobic situation |
| **hypochondriac disorder**  (case 8) | - persistent thinking about the possibility of suffering from a serious and advanced illness - constantly being concerned with the own physical appearance - increased amount of doctor’s consultations - self-diagnostics |
