# Supplementary material for: Sensitivity for multimorbidity: The role of diagnostic uncertainty of physicians when evaluating multimorbid video case-based vignettes
Source: PLoS One. 2019 Apr 10;14(4):e0215049. doi: 10.1371/journal.pone.0215049 (PMC6457556; doi:10.1371/journal.pone.0215049)
Supplement: S2 File — Actor’s scripts for the eight video case-based vignettes and all three sequences (S1 to S3). (DOCX) [file pone.0215049.s002.docx]

**S2 File**

Actor’s scripts for the eight video case-based vignettes and all three sequences (S1 to S3).

**Case 1: food allergy**

S1: The patient is dressed casually (jeans, sweater). He is scratching his neck when he enters the room.

S2: “Over the last few months, as well as yesterday evening, I have been suffering from flatulence and diarrhea. A gastroscopy and colonoscopy have already been done but they showed no results. Sometimes it is stronger and sometimes weaker. But once or twice a week I do have these problems.”

S3: “I noticed that a red spot appears on my arm occasionally. In addition, my tongue feels swollen and breathing frequently becomes more difficult.”

**Case 2: obsessive-compulsive disorder (OCD)**

S1: The patient is wearing a suit and tie; he is adjusting his tie again and again, and stroking his hair. He seems stiff.

S2: “I am failing in my job. In the last six months, I was terminated twice, even though I always did my job very conscientiously. Maybe I was too good. Somehow I am a perfectionist. Nothing is good enough. Sometimes I worry about that.”

S3: “I know that I did a good job but I just have to do everything again to be absolutely sure that my work is good. For example, I had to hand in a report recently. I was already finished but could not hand it in because I had to edit it again and again. I know that this is unreasonable and I try not to do it but I just cannot suppress these thoughts. Yes, that is unpleasant for me.”

**Case 3: arterial hypertension & cardiac insufficiency**

S1: The patient is dressed casually. He is breathing heavily. He has a cigarette pack in his shirt pocket.

S2: “Now and then I suffer from headaches. Sometimes I even get dizzy. Moreover I have the feeling that I gained weight, even though I cannot explain why because I did not change my eating habits.”

S3: “I have not seen a doctor for a long time. I do not care much about any health tips but recently I am really not doing well. When I exert myself, I can feel a pulsation in my head. My legs are frequently swollen but the fact that I have to sleep upright to get enough air bothers me the most.”

**Case 4: arthrosis & hypothyreosis**

S1: The patient is dressed in a sporty outfit. He is limping from the door to the chair and keeps his hand on his thigh.

S2: “I have been doing sports for years, especially marathons. For a few weeks now, my knee has always been swollen after running. The pain is stronger when I have been physically active. That’s why I am not active as much anymore. Perhaps it is because I gained some weight.”

S3: “After getting up in the morning, my knee feels a bit stiff. I have already done physiotherapy but it did not help. In addition I have difficulties warming up and I am often freezing. Sometimes I feel very listless and have difficulties to get going.”

**Case 5: multiple sclerosis & depression**

S1: The patient is dressed casually (jeans, sweater). He is taking little steps and has a waddling gait from the door to the chair.

S2: “Recently everything has become too much for me. More and more I am feeling depressed. Although I am tired, I have difficulties falling asleep. Maybe it is due to the tiredness but sometimes I see as through fog.”

S3: “For a few months I have felt a strange tingling in my hands. It intensifies when I am under stress. It comes and goes. I lost a bit of weight as well.”

**Case 6: diabetes mellitus type 1 & posttraumatic stress disorder (PTSD)**

S1: The patient is dressed casually (jeans, sweater). When the door slams, he startled. He appears tired and exhausted. He is rubbing his eyes and supports his head.

S2: “For some time, I have had recurring nightmares and wake up drenched in sweat at night. Three weeks ago I was ill, and since then I am always thirsty. Because of that, I constantly have to go to the toilet, which is very unpleasant while at work of course.”

S3: “It seems like just yesterday, but it has already been eight months since my girlfriend had a fatal accident. Since then I have lost a few pounds.”

**Case 7: psychotropic substance disorder & panic disorder**

S1: The patient is dressed in a sporty outfit. He wrinkles his forehead and often touches his nose.

S2: “Actually I am a self-confident and successful guy. I often go to parties with my friends and enjoy time relaxing. I am very active during the day as well. I rarely take a break and make sure that I keep busy all the time. But recently I began having these heart palpitations, and it feels like it is hard for me to breathe.”

S3: “Sometimes I have a feeling of I am standing beside myself. Just between us, sometimes I think that I am slowly going mad. To function normally in everyday life I am consuming alcohol more often, not only on weekends as usual.”

**Case 8: social phobia & hypochondriac disorder**

S1: The patient is dressed in a sporty, elegant outfit (shirt and blazer). He appears tense and nervous. He carries a medical reference book with Post-it notes.

S2: “I am sorry for coming here as stressed as I am but I still have to give a presentation later on. I tried to postpone it or to pass it on to a colleague but it did not work out. Anyway, the reason why I came to you is that I want to be examined because I have a malignant brain tumor.”

S3: “I have already seen other doctors but they do not take me seriously. I really feel bad and I am afraid I might vomit during my presentation in front of everyone. I have already observed these symptoms for some time, and I am sure that they can be attributed to the tumor.”
