# Supplementary material for: Sensitivity for multimorbidity: The role of diagnostic uncertainty of physicians when evaluating multimorbid video case-based vignettes
Source: PLoS One. 2019 Apr 10;14(4):e0215049. doi: 10.1371/journal.pone.0215049 (PMC6457556; doi:10.1371/journal.pone.0215049)
Supplement: S4 File — Consent form for all participating physicians to read and to sign. (PDF) [file pone.0215049.s004.pdf]

Universität Zürich[UZH]

ID:__________

Consent form

We investigate the subjective certainty within the diagnostic process of physicians.
I agree to participate in this study „The role of subjective confidence when making a diagnosis in medical practice“. I will be asked to watch video case-based sequences of a patient and to make suspected diagnoses.

I take note that all individuals involved in this research project are bounded to professional discretion. All my data will be used and processed anonymously. Participation in this study is voluntary. I am entitled to interrupt or abandon this study at any time and without having to state a reason.

With my signature I declare that I have read this consent form, and that I have well-understood the content and procedure of this study. I had the possibility to ask comprehension questions, and those had been answered in a satisfactorily manner.

Location and date: ____________________________________

Your signature: ____________________________________

Project leader: Dr. phil. Daniel Hausmann-Thürig, d.hausmann@psychologie.uzh.ch
