# Supplementary material for: Sensitivity for multimorbidity: The role of diagnostic uncertainty of physicians when evaluating multimorbid video case-based vignettes
Source: PLoS One. 2019 Apr 10;14(4):e0215049. doi: 10.1371/journal.pone.0215049 (PMC6457556; doi:10.1371/journal.pone.0215049)
Supplement: S5 File — Written instructions for watching the video case-based vignettes, filling in suspected diagnoses and confidence ratings in a confidence profile after each sequence, as well as filling in short case-related questionnaires and finally a short general questionnaire. (PDF) [file pone.0215049.s005.pdf]

ID:__________

Instruction

Consecutively, you will watch three case-based videos. In each video you will see a fictitious male patient, which tells about his complaints. Each film is subdivided into three sequences. The first sequence shows how the patient enters the room. Therefore you get a first picture of his appearance. In the second and third sequence he verbally informs you about his complaints. After each sequence the film will be stopped. Then you are asked to fill in a confidence profile for the previous sequence. Within the confidence profile you first have to fill in all your suspected diagnoses at this time point in the top of the sheet. You can fill in as much suspected diagnoses as you have. Furthermore, each suspected diagnosis which you have mentioned in one of the sequences before, has to be listed again. When you have done this, you have to mark your subjective confidence level for each suspected diagnosis with a cross. After the third and last sequence of a film you will be asked to additionally fill in a short case-related questionnaire. Having watched all three videos, you finally will be asked to fill in a short general questionnaire.

Below you see an example of an empty confidence profile:

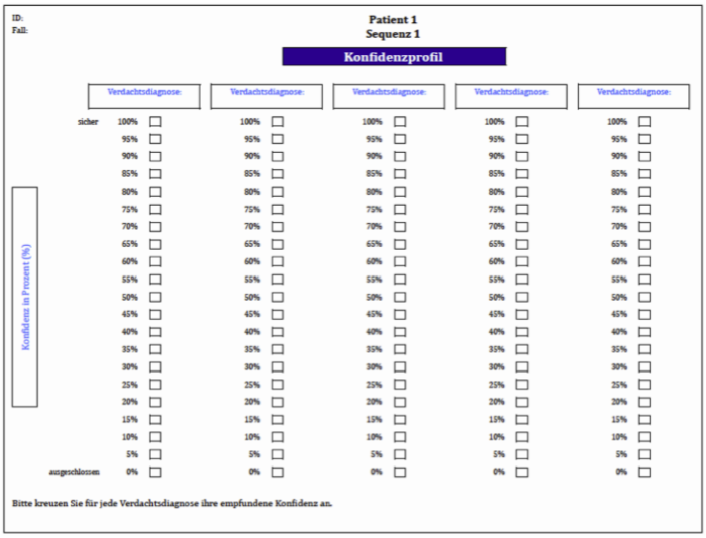
ID:
Fall:

Patient 1
Sequenz 1

Konfidenzprofil

Konfidenz in Prozent (%)

	Verdachtsdiagnose:	Verdachtsdiagnose:	Verdachtsdiagnose:	Verdachtsdiagnose:	Verdachtsdiagnose:
sicher	100% ☐	100% ☐	100% ☐	100% ☐	100% ☐
	95% ☐	95% ☐	95% ☐	95% ☐	95% ☐
	90% ☐	90% ☐	90% ☐	90% ☐	90% ☐
	85% ☐	85% ☐	85% ☐	85% ☐	85% ☐
	80% ☐	80% ☐	80% ☐	80% ☐	80% ☐
	75% ☐	75% ☐	75% ☐	75% ☐	75% ☐
	70% ☐	70% ☐	70% ☐	70% ☐	70% ☐
	65% ☐	65% ☐	65% ☐	65% ☐	65% ☐
	60% ☐	60% ☐	60% ☐	60% ☐	60% ☐
	55% ☐	55% ☐	55% ☐	55% ☐	55% ☐
	50% ☐	50% ☐	50% ☐	50% ☐	50% ☐
	45% ☐	45% ☐	45% ☐	45% ☐	45% ☐
	40% ☐	40% ☐	40% ☐	40% ☐	40% ☐
	35% ☐	35% ☐	35% ☐	35% ☐	35% ☐
	30% ☐	30% ☐	30% ☐	30% ☐	30% ☐
	25% ☐	25% ☐	25% ☐	25% ☐	25% ☐
	20% ☐	20% ☐	20% ☐	20% ☐	20% ☐
	15% ☐	15% ☐	15% ☐	15% ☐	15% ☐
	10% ☐	10% ☐	10% ☐	10% ☐	10% ☐
	5% ☐	5% ☐	5% ☐	5% ☐	5% ☐
ausgeschlossen	0% ☐	0% ☐	0% ☐	0% ☐	0% ☐

Bitte kreuzen Sie für jede Verdachtsdiagnose ihre empfundene Konfidenz an.

Project leader: Dr. phil. Daniel Hausmann-Thürig, d.hausmann@psychologie.uzh.ch
