# Supplementary material for: Sensitivity for multimorbidity: The role of diagnostic uncertainty of physicians when evaluating multimorbid video case-based vignettes
Source: PLoS One. 2019 Apr 10;14(4):e0215049. doi: 10.1371/journal.pone.0215049 (PMC6457556; doi:10.1371/journal.pone.0215049)
Supplement: S6 File — Empty template for filling in suspected diagnoses and confidence ratings after each sequence of a video. (PDF) [file pone.0215049.s006.pdf]

ID: _____

Case: _____

Patient 1 **Sequence 1**

Confidence profile

Subjective confidence level in percent (%)

	Suspected diagnosis:	Suspected diagnosis:	Suspected diagnosis:	Suspected diagnosis:	Suspected diagnosis:
sure	100% ☐	100% ☐	100% ☐	100% ☐	100% ☐
	95% ☐	95% ☐	95% ☐	95% ☐	95% ☐
	90% ☐	90% ☐	90% ☐	90% ☐	90% ☐
	85% ☐	85% ☐	85% ☐	85% ☐	85% ☐
	80% ☐	80% ☐	80% ☐	80% ☐	80% ☐
	75% ☐	75% ☐	75% ☐	75% ☐	75% ☐
	70% ☐	70% ☐	70% ☐	70% ☐	70% ☐
	65% ☐	65% ☐	65% ☐	65% ☐	65% ☐
	60% ☐	60% ☐	60% ☐	60% ☐	60% ☐
	55% ☐	55% ☐	55% ☐	55% ☐	55% ☐
	50% ☐	50% ☐	50% ☐	50% ☐	50% ☐
	45% ☐	45% ☐	45% ☐	45% ☐	45% ☐
	40% ☐	40% ☐	40% ☐	40% ☐	40% ☐
	35% ☐	35% ☐	35% ☐	35% ☐	35% ☐
	30% ☐	30% ☐	30% ☐	30% ☐	30% ☐
	25% ☐	25% ☐	25% ☐	25% ☐	25% ☐
	20% ☐	20% ☐	20% ☐	20% ☐	20% ☐
	15% ☐	15% ☐	15% ☐	15% ☐	15% ☐
	10% ☐	10% ☐	10% ☐	10% ☐	10% ☐
	5% ☐	5% ☐	5% ☐	5% ☐	5% ☐
excluded	0% ☐	0% ☐	0% ☐	0% ☐	0% ☐

Please mark your subjective confidence level for each suspected diagnosis with a cross
