# Supplementary material for: Sensitivity for multimorbidity: The role of diagnostic uncertainty of physicians when evaluating multimorbid video case-based vignettes
Source: PLoS One. 2019 Apr 10;14(4):e0215049. doi: 10.1371/journal.pone.0215049 (PMC6457556; doi:10.1371/journal.pone.0215049)
Supplement: S7 File — Empty sheet for filling in additional information about suspected diagnoses, reference of patient, difficulty of diagnosis, and missing additional information after each video. (PDF) [file pone.0215049.s007.pdf]

Case-related Questionnaire

Which suspected diagnosis/diagnoses would you make?

1. ______________________________
2. ______________________________
3. ______________________________
4. ______________________________
5. ______________________________

further ______________________________

Would you refer this patient?

no ☐ yes ☐ **If yes, to whom?** ________________

How difficult was it to make a diagnosis?

very easy	easy	moderate	difficult	very difficult
☐	☐	☐	☐	☐

Did you have missed additional information?

yes ☐ no ☐

If yes, which one?

☐ further symptoms

☐ medical tests

☐ subjective information (e.g. posture, skin color, etc.)

☐ possibility for further inquiries

☐ other ______________________________

ID: __________ **Case**: __________ **Sequence**: __________

Project leader: Dr. phil. Daniel Hausmann-Thürig, d.hausmann@psychologie.uzh.ch
