# Supplementary material for: Sensitivity for multimorbidity: The role of diagnostic uncertainty of physicians when evaluating multimorbid video case-based vignettes
Source: PLoS One. 2019 Apr 10;14(4):e0215049. doi: 10.1371/journal.pone.0215049 (PMC6457556; doi:10.1371/journal.pone.0215049)
Supplement: S8 File — Empty sheet for filling in additional information about the difficulty of making a diagnosis, case experience and difficulty, and missing additional information at the end of participation. (PDF) [file pone.0215049.s008.pdf]

GQ

ID: ___________

General Questionnaire

How difficult was it to make a diagnosis?

very easy	easy	moderate	difficult	very difficult
☐	☐	☐	☐	☐

Did you perceive one of the three cases as easier compared to the others?

no ☐ yes ☐

If yes, which one?

☐ the first one

☐ the second one

☐ the third one

Did you perceive one of the three cases as more difficult compared to the others?

no ☐ yes ☐

If yes, which one?

☐ the first one

☐ the second one

☐ the third one

At which point your subjective confidence rating was elevated versus reduced?

if elevated?

☐ when I knew this case from literature

☐ when I knew this case from practical experience

☐ other ____

if reduced?

☐ when the number of symptoms was too high versus low

☐ when I didn’t know this case from practical experience

☐ other ____

Did you generally have missed additional information?

yes ☐ no ☐

If yes, which one?

- ☐ further symptoms
- ☐ medical tests
- ☐ subjective information (e.g. posture, skin color, etc.)
- ☐ possibility for further inquiries
- ☐ other ______________________________

How many of your patients are multimorbid? (please have a guess)

< 25%	up to 25%	up to 50%	up to 75%	> 75%
☐	☐	☐	☐	☐

How realistic was this experiment?

absolutely realistic	very realistic	moderate realistic	rather not realistic	not at all realistic
☐	☐	☐	☐	☐

Comments:

....

....

....

....

Date: ______________________________

Cases: ______________________________

Gender: ______________________________

Project leader: Dr. phil. Daniel Hausmann-Thürig, d.hausmann@psychologie.uzh.ch
