# Supplementary material for: Sensitivity for multimorbidity: The role of diagnostic uncertainty of physicians when evaluating multimorbid video case-based vignettes
Source: PLoS One. 2019 Apr 10;14(4):e0215049. doi: 10.1371/journal.pone.0215049 (PMC6457556; doi:10.1371/journal.pone.0215049)
Supplement: S1 Table — Descriptive statistics for all eight video case-based vignettes (for abbreviations see Table 1). (DOCX) [file pone.0215049.s009.docx]

**S1 Table**

Descriptive statistics for all eight video case-based vignettes (for abbreviations see Table 1).

|  | **Case 1**  M^s^ | **Case 2**  M^m^ | **Case 3**  MM^ss-r^ | **Case 4**  MM^ss-u^ | **Case 5**  MM^sm-r^ | **Case 6**  MM^sm-u^ | **Case 7**  MM^mm-r^ | **Case 8**  MM^mm-u^ |
| --- | --- | --- | --- | --- | --- | --- | --- | --- |
| **Number of cases / per physician** | **38** | **60** | **13** | **41** | **27** | **46** | **10** | **34** |
| GPs (*N* = 28) | 13 | 15 | 13 | 16 | 15 | 12 | 0 | 0 |
| Residents (*N* = 25) | 25 | 25 | 0 | 25 | 0 | 25 | 0 | 25 |
| Psychiatrists (*N* = 21) | 0 | 20 | 0 | 0 | 12 | 9 | 10 | 9 |
| Mean age in years / (*SD*) | 39.03  (11.82) | 46.40 (13.80) | 54.15 (8.23) | 39.71 (11.96) | 55.85 (8.30) | 43.35 (13.90) | 57.10 (8.12) | 39.03 (12.85) |
| Gender (female in %) | 42.1 | 43.3 | 23.1 | 60.0 | 33.3 | 41.3 | 50.0 | 52.9 |
| Median work experience in years / [*Interquartile range, Q1–Q3 ]* | 5.0  [3.1–16.5] | 19.5  [4.0–29.3] | 24.0  [21.0–32.0] | 6.0  [3.5–18.0] | 27.0  [20.0–35.0] | 6.5  [3.6–25.8] | 28.0  [20.5–34.5] | 5.0  [3.0–20.5] |
| Median case experience in cases / [*Interquartile range, Q1–Q3]* | 11.0  [3.0–21.0] | 7.0  [2.5–21.0] | 21.0  [21.0–50.0] | 11.0  [3.0–21.0] | 50.0  [21.0–50.0] | 7.0  [3.0–21.0] | 21.0  [13.5–50.0] | 3.0  [3.0–21.0] |
| Distribution of classified diagnoses |  |  |  |  |  |  |  |  |
| Number of “monomorbid” diagnoses | **15** | **46** | 5 | 25 | 20 | 22 | 4 | 19 |
| Number of “multimorbid” diagnoses | 0 | 4 | **6** | **5** | **3** | **11** | **5** | **8** |
| Number of “no diagnosis” | 23 | 10 | 2 | 11 | 4 | 13 | 1 | 7 |
| Total number of (different)  mentioned ICD-10 diagnoses | 70 | 57 | 29 | 64 | 40 | 58 | 15 | 43 |
| **Number of *accurate* patterns** | **12** | **49** | **10** | **27** | **23** | **29** | **8** | **27** |
| multimorbid (hit/totally correct) |  |  | 4 | 3 | 3 | 5 | 4 | 8 |
| multimorbid (partially correct) | 0 | 4 | 2 | 2 | 0 | 4 | 1 | 0 |
| monomorbid (miss) |  |  | 4 | 22 | 20 | 20 | 3 | 19 |
| monomorbid (correct rejection) | 12 | 45 |  |  |  |  |  |  |
| Mean rated realism / (*SD*) | 3.16 (0.82) | 3.35 (0.86) | 3.46 (0.88) | 3.15 (0.85) | 3.59 (0.57) | 3.17  (0.93) | 3.80 (0.92) | 3.21 (0.84) |
| Mean rated difficulty / (*SD*) | 3.68 (0.71) | 2.55 (0.85) | 2.62 (0.65) | 3.20 (0.82) | 2.70 (0.99) | 2.84 (0.85) | 2.20 (0.79) | 2.50 (0.93) |
